# A Causal Analysis of the Effect of Age and Sex Differences on Brain Atrophy in the Elderly Brain

**DOI:** 10.3390/life12101586

**Published:** 2022-10-12

**Authors:** Jaime Gómez-Ramírez, Miguel A. Fernández-Blázquez, Javier J. González-Rosa

**Affiliations:** 1 Department of Psychology, University of Cadiz, 11003 Cadiz, Spain; 2 Institute of Biomedical Research Cadiz (INiBICA), 11009 Cadiz, Spain; 3 Department of Biological and Health Psychology, Universidad Autónoma de Madrid, 28049 Madrid, Spain

**Keywords:** MRI, Causal inference, brain atrophy, MCMC sex differences, probabilistic Bayesian modelling

## Abstract

We studied how brain volume loss in old age is affected by age, the APOE gene, sex, and the level of education completed. The quantitative characterization of brain volume loss at an old age relative to a young age requires—at least in principle—two MRI scans, one performed at a young age and one at an old age. There is, however, a way to address this problem when having only one MRI scan obtained at an old age. We computed the total brain losses of elderly subjects as a ratio between the estimated brain volume and the estimated total intracranial volume. Magnetic resonance imaging (MRI) scans of 890 healthy subjects aged 70 to 85 years were assessed. A causal analysis of factors affecting brain atrophy was performed using probabilistic Bayesian modelling and the mathematics of causal inference. We found that both age and sex were causally related to brain atrophy, with women reaching an elderly age with a 1% larger brain volume relative to their intracranial volume than men. How the brain ages and the rationale for sex differences in brain volume losses during the adult lifespan are questions that need to be addressed with causal inference and empirical data. The graphical causal modelling presented here can be instrumental in understanding a puzzling scientific area of study—the biological aging of the brain.

## 1. Introduction

Historically, the investigation of brain volume variations with age can be classified into at least three well-defined periods: the era of autopsies, followed by the utilization of magnetic resonance imaging (MRI), up to the present time, which is dominated by a computational anatomy approach, making use of MRI leveraged with data-analytical methods.

Early evidence of the effect of ageing on the brain size and structure comes from autopsy studies conducted in the 19th century that indicated that brain weight decreased slowly but surely with age [1,2]. Autopsies helped to solidify the commonly held belief that brain weight is stable between 20 and 50 and progressively decays thereafter. Large-sample autopsy-based studies, still prior to the MRI era, suggested that brain weight reached its maximum in the late teens and declined very slowly (0.1–0.2% a year) up to the 60 s and 70 s, after which the decline became faster [3,4]. In a 1980 study [5], the weights of fresh brains from autopsies of 1261 subjects aged from 25 to 80 showed that the brain mass decreased rapidly after the age of 80. It also indicated different atrophy patterns based on ethnicity and sex. Around the same time, brain autopsies showed that a progressive decline in brain weight begins at approximately 45 to 50 years of age and reaches its lowest values after 86 [6]. The authors of the study postulated that the maximum brain weight attained in young adults is reached at 19 years of age, estimating an accumulated loss of brain weight of 11% between the ages 19 and 86 and detecting differential rates of change in brain weight depending on age and less so on sex. However, studies based on autopsies present problems of reliability and selection bias, and, most importantly, they cannot tell us anything about cerebral atrophy in living individuals. The advent of noninvasive imaging changed this.

Magnetic resonance imaging—and, before that, computed tomography—created the possibility of noninvasively and repeatedly measuring the cerebral volume in vivo [7]. Imaging studies revealed global volume losses and regional variations as major effects of ageing on the brain. Nevertheless, the estimates of volume and tissue losses required manual outlining and the a priori selection of brain areas [8,9].

The advent of new computerized methods that are sensitive to variations in the size, shape, and tissue characteristics of brain structures represent the most recent stage in the study of brain anatomy in ageing, offering a new set of tools that were unknown to previous researchers who needed to rely upon autopsies and the manual outlining of MRI and tomographies [10]. Specifically, the game-changer event was the development of voxel-based morphometry (VBM), a whole-brain technique for characterizing regional cerebral volume and tissue concentration differences in structural magnetic resonance images. MRI studies with automatic segmentation of ageing brains in vivo have proliferated since then.

There is growing evidence that age has a stronger influence on brain structure in older patients than it does in younger adults, but the onset and the type of decline (linear or nonlinear) depend on the tissue and brain region [11,12]. The common understanding of tissue atrophy indicates that the onset of grey matter atrophy may occur in young adulthood, at approximately 18. White matter, on the other hand, remains relatively stable until old age. Although there is no theory of human brain ageing available that is capable of making robust predictions about brain growth and atrophy, we know that rapid growth occurs during childhood/adolescence, with a particularly dramatic growth rate during the first 3 months, at approximately 1% per day, reaching half of the adult brain volume by the end of the first 3 months [13]. Between 18 and 35 years old, the brain experiences a period of consolidation with no significant brain tissue loss. After 35 years, Hedman and colleagues [14] suggested that a steady volume loss of 0.2% per year occurs, which accelerates gradually to an annual brain volume loss of 0.5% by age 60. After 60, the same study indicated a steady volume loss of more than 0.5% per year.

Fjell et al. [15] observed a nonlinear decline across chronological age in the hippocampus and caudate, but they observed linear decline slopes for the thalamus and accumbens. In [16], cortical thinning was found to be significantly altered by hypertension and apolipoprotein-Ee4 (APOEe4), with frontal and cingulate cortices thinning more rapidly in APOEe4 carriers. Additional longitudinal studies have found different brain atrophy patterns according to clinical conditions, including cognitive decline and Alzheimer’s disease [17,18,19] and multiple sclerosis [20]. Nonetheless, the small sample size and the lower reliability of the segmentation of small structures are recognized caveats in longitudinal studies [21].

A number of studies have used a ratio of brain volume to estimate the total intracranial volume in the context of studying ageing and sex diphormism [22,23]. The total intracranial volume has been used as a correction factor for head size variability when assessing total brain volume [24] and as a covariate in regression analysis to investigate the role played by sex in neuroanatomical volume differences [25]. There is contradictory evidence regarding sexual dimorphism in neuroanatomical structures [26,27], and the differences in the volume found could be attributed to the intracranial volume normalization method used [28].

In the machine-learning literature, brain age is understood as the model estimate to be compared with the given or chronological age [29]. However, this approach depends on the model’s capacity to accurately predict the brain’s biological age. New cellular and molecular approaches to brain senescence and decline, such as transcriptome profiling [30], DNA methylation [31], and immune metrics such as the inflammatory clock of ageing (iAge) [32], represent opportunities to understand the divergence between biological age and chronological age.

In this work, we depart from the usual correlational and linear regression approach to assessing sex- and age-related differences in brain volume. Here, we used directed acyclic graphs [33] to investigate whether there is a direct causal relationship between sex and brain atrophy in normal elderly subjects. We approximated brain atrophy as the ratio between the brain volume and a proxy of the brain’s maximum size reached at some point at a young age.

By quantifying, albeit approximately, the brain volume loss at an older age relative to its upper bound at a young age, we can make educated guesses about the effect of brain ageing in a person. The mismatch between the actual brain volume and the expected brain volume according to the person’s age may contain valuable information, enabling us to better understand brain ageing dynamics.

## 2. Methods

### 2.1. Study Participants

The dataset used here came from a single-centre, observational cohort study of 1213 subjects [29,34,35]. The participants were home-dwelling elderly volunteers, aged 69 to 85, without relevant psychiatric, neurological, or systemic disorders. Of the initial 1213 subjects, those who were diagnosed with MCI or dementia, plus those who lacked a brain MRI, were excluded from our analysis, resulting in a cohort of 890 healthy elderly subjects. After signing informed consent forms, the participants underwent a yearly systematic clinical assessment, including medical history evaluations, neurological and neuropsychological exams, blood collection, and brain MRI.

Ethical approval was granted by the Research Ethics Committee of the Instituto de Salud Carlos III (CEI PEI 46 2011-v2014), and written informed consent was obtained from all of the participants. The authors assert that all procedures contributing to this work comply with the ethical standards of the relevant national and institutional committees on human experimentation and with the Helsinki Declaration of 1975 and its later amendments.

The ordinal encoding system of educational attainment was as follows: 0—no formal education, 1—primary education, 2—middle or high school degree, and 3—university degree. Cognitive status was determined with the Mini-Mental State Examination (MMSE), the Free and Cued Selective Reminding Test (FCSRT), semantic fluency testing, the Digit-Symbol Test, and the Functional Activities Questionnaire (FAQ). Memory loss is among the first and most important symptoms of patients suffering from Alzheimer’s disease (AD) and mild cognitive impairment (MCI), and although it is not uncommon to refer to the type of neuropsychological assessment performed in this study as a memory test, cognitive status is a more suitable term. For a more detailed description of the neuropsychological protocols used to assess the cognitive function of the participants in this study, see [36].

The APOE genotype was studied using total DNA isolated from peripheral blood following standard procedures. The APOE variable was coded 1 for the presence of at least one *e*4 carrier and 0 for noncarriers. A family history of AD was coded as 0 for subjects with no parents or siblings diagnosed with dementia and 1 for those with at least one parent or sibling diagnosed with dementia.

### 2.2. MRI Data Acquisition and Preprocessing

**Figure 1:**
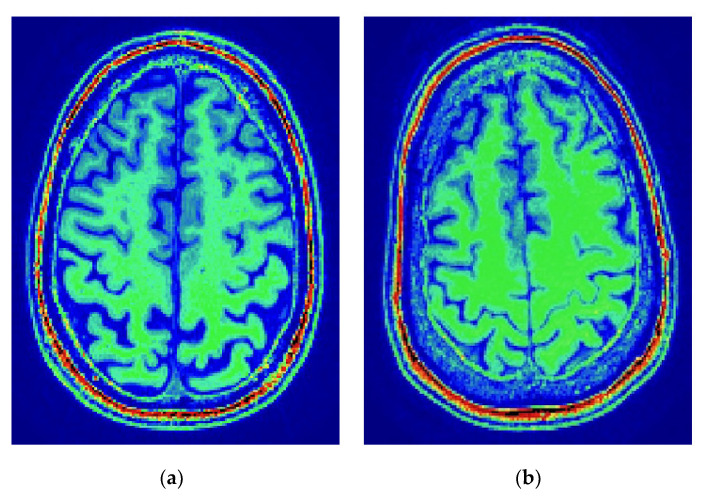
Axial views of the brain and their membranous envelopes obtained from two subjects in this study. The Brain2ICV or brain-volume-to-intracranial-volume ratio was Brain2ICV = TBV/eTIV = 929,035/1,460,465 = 0.7196 for the left figure, and Brain2ICV = TBV/eTIV = 929,035/132,7593 = 0.6997 for the right figure. (**a**) Male subject, 74 years old, Brain2ICV = 71.96%; (**b**) Female subject, 76 years old, Brain2ICV = 69.97%.

The imaging data were acquired in the sagittal plane on a 3T General Electric scanner (GE Milwaukee, Milwaukee, WI, USA), utilizing T1-weighted inversion recovery, a supine position, a flip angle of 12°, a 3-D pulse sequence: echo time Min. full, a time inversion of 600 ms, a receiver bandwidth of 19.23 kHz, a field of view of 24.0 cm, a slice thickness of 1 mm, and freq phase 288. The brain volume loss at the moment of MRI compared to the maximum brain volume was computed as the brain segmentation volume compared to the estimated total intracranial volume (eTIV) [37] as a ratio (ICV and eTIV, the FreeSurfer term for intracranial volume, are used equivalently). ICV and eTIV are used interchangeably. The postprocessing was performed with FreeSurfer [38,39], version freesurfer-darwin-OSX-ElCapitan-dev-20190328-6241d26, running on a Mac OS X, product version 10.14.5. For the sake of illustration, Figure 1 shows the results produced regarding the intracranial volume segmentation for two subjects in the study.

The total intracranial volume acts as a scaffolding of the brain and sets an upper bound for the brain’s volume. Accordingly, it is possible to build a proxy of the brain atrophy that an elderly person went through in their adult life by calculating the ratio between the brain volume (TBV) and the total intracranial volume (eTIV), which represents the upper limit of the brain volume. Thus, the ratio of the brain volume to the intracranial volume is defined as Brain2ICV = TBV/eTIV.

The estimated intracranial volume (eTIV) obtained from FreeSurfer is not based on the direct segmentation of all structures within the skull demarcation; rather, its estimation depends on the alignment between the T1 skull boundaries and the average brain based on 305 T1-weighted MRI scans [40,41]. Measurements of total brain volume (TBV) with FreeSurfer are robust across field strengths [42], and variations in individual head size are corrected in the assessment of brain volume losses at an older age relative to their upper bound at a young age (Brain2ICV) by normalizing them against the total intracranial volume [43].

### 2.3. Statistical Data Analysis

Table 1 includes a description of the variables considered in this study, providing the mean and the standard deviation for the continuous variables—age, cognitive test score, and the brain volume to intracranial volume ratio (Brain2ICV)—and the classes, together with the number of elements for each class, for the categorical variables—sex, APOE, family history of AD, and school level. To assess the strength of the linear association between Brain2ICV and the predictor variables, we performed Pearson’s correlation, point biserial correlation, and an analysis of variance, depending on whether the variable was continuous, dichotomous (as in the case of sex, family history of AD, and APOE), or discrete with more than two values (as in the case of school level). A hypothesis test was performed to study the significance of the Pearson’s correlation coefficient.

### 2.4. Causal Data Analysis

Correlation is the degree to which two variables show a tendency to vary together. Causality, on the other hand, is the relationship between an observed effect and what caused it.

For variable *C* to cause another variable *E*, (*C→E*), there must be a flow of information from cause *C* to effect *E*. Here, we intended to identify the causal paths built on top of the correlation paths that link one or more causes with an effect, specifically, the variables that causally affect Brain2ICV. Thus, we aimed to study the causal connections between the correlated variables using probabilistic Bayesian modelling [44] and the mathematics of causal inference proposed by Pearl [45].

Bayesian model selection aims to compute the posterior distribution, which contains all of the information needed regarding the model parameters. The posterior distribution also allows us to generate predictions based on the actual data and the estimated parameters. Once we have the posterior distribution, we can make predictions, *y*ˆ, based on data, *y*, and the estimated parameters, *θ*.

In Bayesian inference, the highest density interval (HDI) is the summary credible interval for the posterior distribution. Thus, all *θ* values inside the HDI have a higher probability density, i.e., credibility, than any value outside the HDI. For example, 95% HDI defines an interval spanning 95% of the distribution so that every point inside the interval has higher credibility than any point outside the interval and the total probability of all such *θ* values is 95%. The posterior predictive distribution is an average of the conditional predictions over the posterior distribution of *θ* (Equation (1)):

(1) $$p(\hat{y}|y)\;=\;p(\hat{y}|\theta )p(\theta |y)d\theta$$

Bayesian networks are probabilistic graphical models that represent the dependencies of a set of variables and their joint distribution [46]. Specifically, a Bayesian network is a graph with directed edges (associations) and with an acyclic structure, that is, a node cannot be its own ancestor or descendant. The underlying causal graph structure provides qualitative information about the conditional independencies of the variables of interest.

Directed acyclic graphs, or DAGs for short, are used to model a priori causal assumptions [47]. Each node represents a random variable, and the arrows represent directed causal paths, i.e., the potential flows of causation among the nodes. DAGs provide a graphical representation of the causal relationships between variables as postulated by the modeller. Accordingly, causal analysis requires one to postulate up front, before the process that generates the data, that is, it requires the modeller’s input, which must always be informed by empirical evidence. The identification of causal associations will consequently depend on the model’s complexity. Causal graphs are incrementally being used, for example, in modern epidemiology to assess causal effects, allowing for the intuitive interpretation of the underlying data-generating process [48,49].

**Figure 2:**
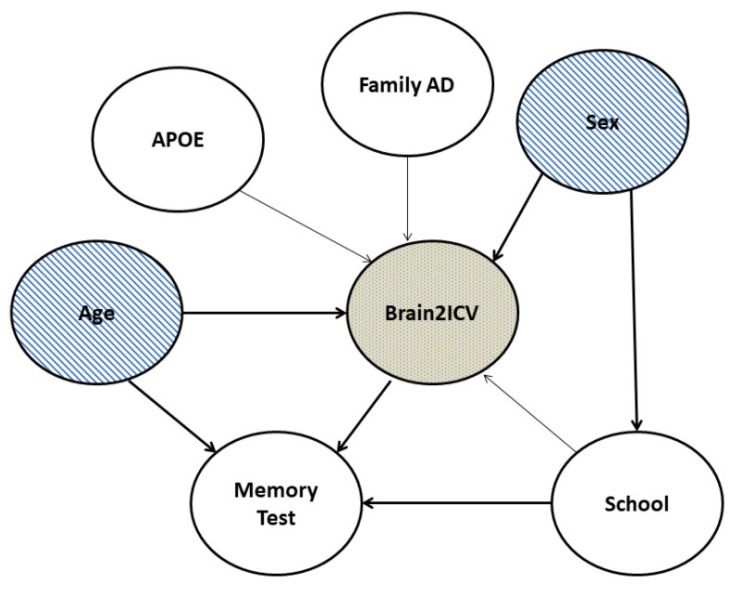
Directed acyclic graph (DAG) that postulates causal relationships between the variables in the study. Variable C in a causal diagram can only causally affect variable E when there is a direct path from C to E. Based on the DAG, sex directly influenced Brain2ICV, school level and age directly influenced Brain2ICV, and memory and school level directly influenced memory. The term “memory test” needs to be understood as an abridged version of the neuropsychological cognitive assessments defined in Section 2.1.

Figure 2 shows the DAG used to model the causal relationships between the variables in this study. The DAG expresses the recognition that a large part of the structure of causal inference is derived from multivariate relationships. A causal model of two variables—for example, age and brain atrophy—needs to be complemented with additional factors of interest, notably sex. Furthermore, the notion that age causes changes in the brain volume, vasculature, and cognition is uncontested [50,51].

The causal graph structure shown in Figure 2 provides qualitative information about the conditional independencies of the variables of interest. We are interested in clarifying the causal structure in the coloured nodes depicted in Figure 2, that is, how sex and age affect Brain2ICV. Sex and age were, according to the DAG, the only two relevant variables that directly influenced Brain2ICV. It is worth remarking that a DAG cannot be directly generated from observational data alone; the structure of the DAG makes use of expert knowledge. Once the DAG is in place, it can be used to guide interventions that substantiate the causal reasoning that emanates from the DAG.

Although the association between age and brain atrophy is indisputable, the question of how sex mediates brain atrophy is unclear, with studies suggesting that the ratio of the total brain volume to intracranial brain volume is higher either in men [27] or in women [25]. The question we aimed to answer can be expressed as follows: Is there a direct causal relationship between sex and Brain2ICV?

As previously stated, causality cannot be answered through the use of data alone, and a model of the process that generated the data is required. To investigate the relationship between sex and Brain2ICV, we built the probabilistic Bayesian model expressed in Equations (2a)–(2d):

*B_i_∼ N(µ_i_, σ)* (2a)

*µ_i_ = γ_X[i]_* (2b)

*γ_j_∼ N*(0, 1), for *j* = 1, 2 (2c)

*σ∼* Hal f Normal(1) (2d)

where *B_i_* denotes the variable Brain2ICV or the ratio between the brain volume at an old age and the maximum brain volume at a young age for Subject *i*, and this is normally distributed with a mean *µ_i_* and a standard deviation *σ*. The index variable for sex *γ_j_*, with the index *j* = 1, 2, represents the average of Brain2ICV for male (*j* = 1) and female (*j* = 2) subjects (no order implied), which are normally distributed using the same prior, *N*(0, 1), for both male and female subjects [52]. The prior *σ* is assumed to be normally distributed, half-normally to be exact, with a standard deviation equal to 1 (a half-normal distribution can be directly sampled from a normal distribution by taking the absolute value of each sampled value).

## 3. Results

We first present the results of the statistical and correlation analysis in Section 3.1. Then, in Section 3.2, we describe the causal inference results using probabilistic programming and causal diagrams.

### 3.1. Statistical and Correlation Analysis

**Figure 3:**
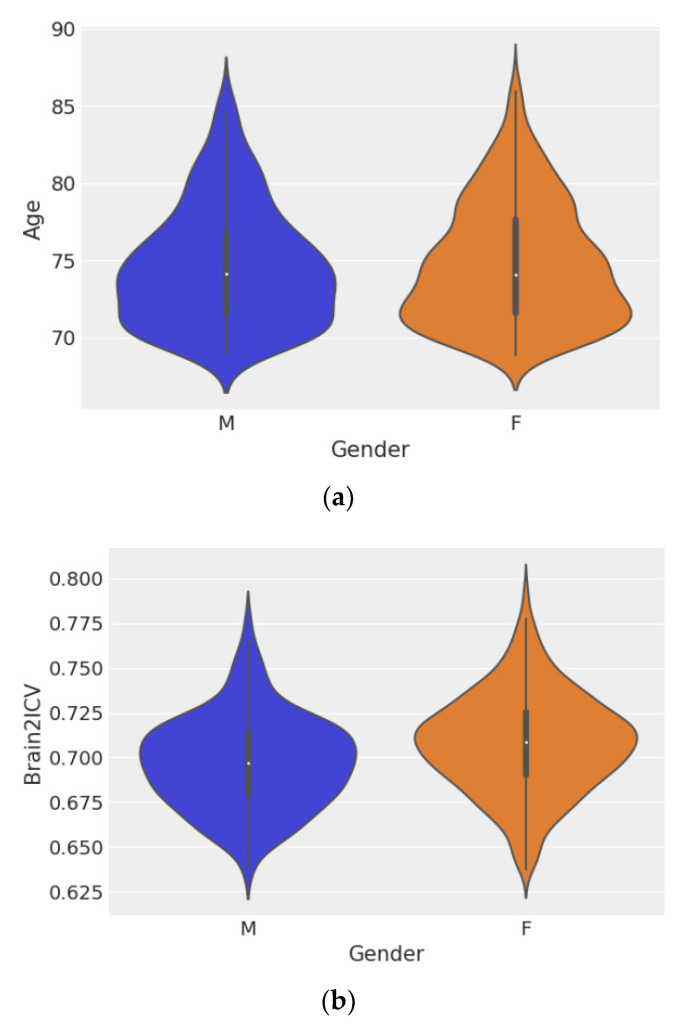
Violin plots of sex distribution (left) and Brain2ICV grouped by sex (right). The *t*-test for the means of the two independent samples of scores composed of the ages of men and women showed no difference between the groups (*p* = 0.572). (**a**). The *t*-test for the means of the Brain2ICV of men and women showed a *p value* of 2.583−8 *<* 0.01 (**b**). (**a**) Plot of the ages of the participants grouped by sex. The distribution of age was *µ*_*M*_ ± *σ*_*M*_ = 74.64 ± 3.83 for men and *µ*_*F*_ ± *σ*_*F*_ = 74.79 ± 3.91 for women. (**b**) Plot of Brain2ICV grouped by sex. The Brain2ICV distribution for men was *µ*_*M*_ ± *σ*_*M*_ = 0.697 ± 0.026, and for women, it was *µ*_*F*_ ± *σ*_*F*_ = 0.708 ± 0.028.

Figure 3 shows violin plots of age grouped by sex (Figure 3a) and Brain2ICV grouped by sex (Figure 3b). Hypothesis testing of the ages of men and women showed no difference between the two groups, with *p* = 0.572 (Figure 3a). However, the test for the means of the Brain2ICV values for men and women (Figure 3b) yielded *p* = 2.583^−8^, which is less than the threshold of 1%, thus disproving the null hypothesis. According to Figure 3b, women, on average, entered old age (70 or older) with slightly less brain atrophy than men, as explained by the brain-to-intracranial-volume ratio variable (Brain2ICV).

**Figure 4:**
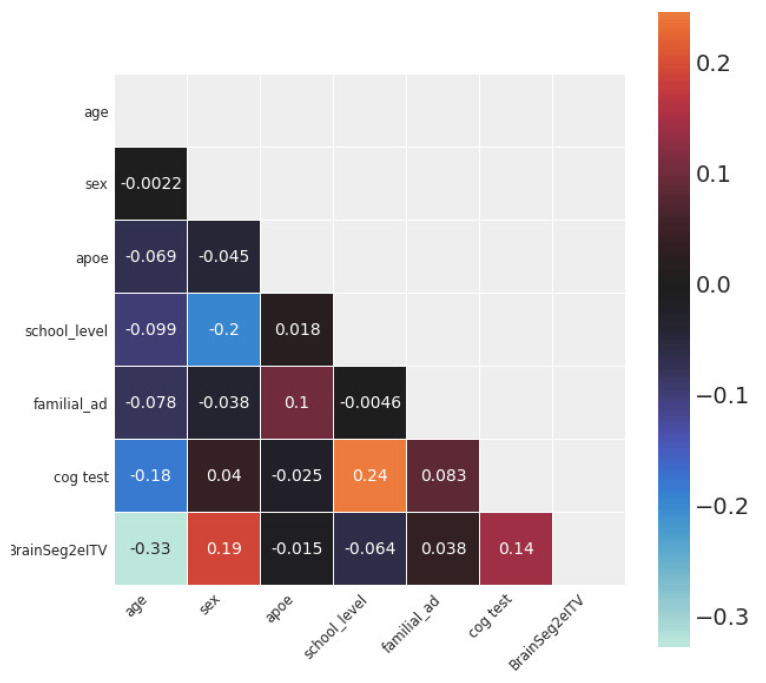
Correlation matrix of the variables used in this study. The variable of interest, brain volume to intracranial volume (Brain2ICV), is depicted in the last row. Age showed the strongest linear correlation with Brain2ICV (*ρ* = −0.33), followed by sex (*ρ* = 0.19) and memory test score (*ρ* = 0.14). School level, APOE, and family history of AD showed no correlation with Brain2ICV.

Figure 4 shows the statistical dependence between every pair of variables in the study. The variables with the largest Pearson’s correlation coefficients with Brain2ICV were age (*ρ* = −0.33), sex (*ρ* = 0.19), and memory score test results (*ρ* = 0.14).

Table 2 shows the analysis of variance (ANOVA) conducted with a linear ordinary least squares (OLS) model [53]. We were interested in the variables that may have an effect on Brain2ICV, which, according to the DAG depicted in Figure 2, were age, APOE, family history of AD, sex, and school level. According to Table 2, both age and sex had p values less than the significance level of 1%; therefore, the null hypothesis—that age and sex have no effect on the brain-to-ICV ratio—can be rejected. The variable cognitive test was not included since we were interested in variables that potentially caused Brain2ICV, that is to say, the directionality of the arrow must be directed towards Brain2ICV.

### 3.2. Causal Analysis

To answer the question, Is there a direct causal relationship between sex and Brain2ICV?, we proceeded by studying the difference in Brain2ICV values between the male and female groups. We are thus interested in the difference between the two groups, rather than in the expected brain atrophy for each sex group, which was already shown in Figure 3b. To compute this contrast, we used samples from the posterior distribution; that is, we fit the model shown in Equations (2a)–(2d) to the data to have access to the posterior distribution of the difference or the contrast between the male and female groups.

Table 3 shows the posterior distribution of the three parameters declared in Equations (2a)–(2d) (*µ*_1_, *µ*_2_, *σ*) and the posterior of the difference between the mean brain atrophy values between the male group and the female group, or *µ*_1_ − *µ*_2_.

The interpretation of the parameters in Table 3 is straightforward; the mean and standard deviation of the posterior distribution of Brain2ICV in men were 0.697 ± 0.002, whereas for women, these were 0.708 ± 0.001. More importantly, the difference between the posterior distribution of the means showed that women entered old age with approximately 1% less atrophy than men, 0.011 ± 0.002. The high posterior density interval (HDI) was always negative; that is, when comparing the distribution of men and women, the area of the distribution of the Brain2ICV results for women was larger than that of men. Note that the HDI is not the same as a confidence interval. HDI is the probability of a variable having some value, whereas a frequentist confidence interval contains or does not contain the true value of a parameter since, in the frequentist philosophy, parameters are treated as nonrandom objects [54]. The kernel density estimates (KDE) of the Bayesian posterior of parameters *µ*_1_, *µ*_2_, *and σ*, as well as the model evaluation, are provided in the Supplementary Materials (Figures S1–S4).

Previous correlational brain volumetric studies have suggested sex differences [28], with women having generally larger volumes after adjusting for total intracranial volume [25]. Our results, for the first time, show brain volumetric diphormism in the ageing brain using Bayesian statistical inference and posterior analysis, rather than point estimates [55,56].

Now we are in a position to affirmatively answer the question posed above. Sex has a direct effect on Brain2ICV. The expected difference or contrast between the sample means of female and male brains in relation to intracranial volume (Brain2ICV) shows that Brain2ICV was larger for women.

## 4. Discussion

**Figure 5:**
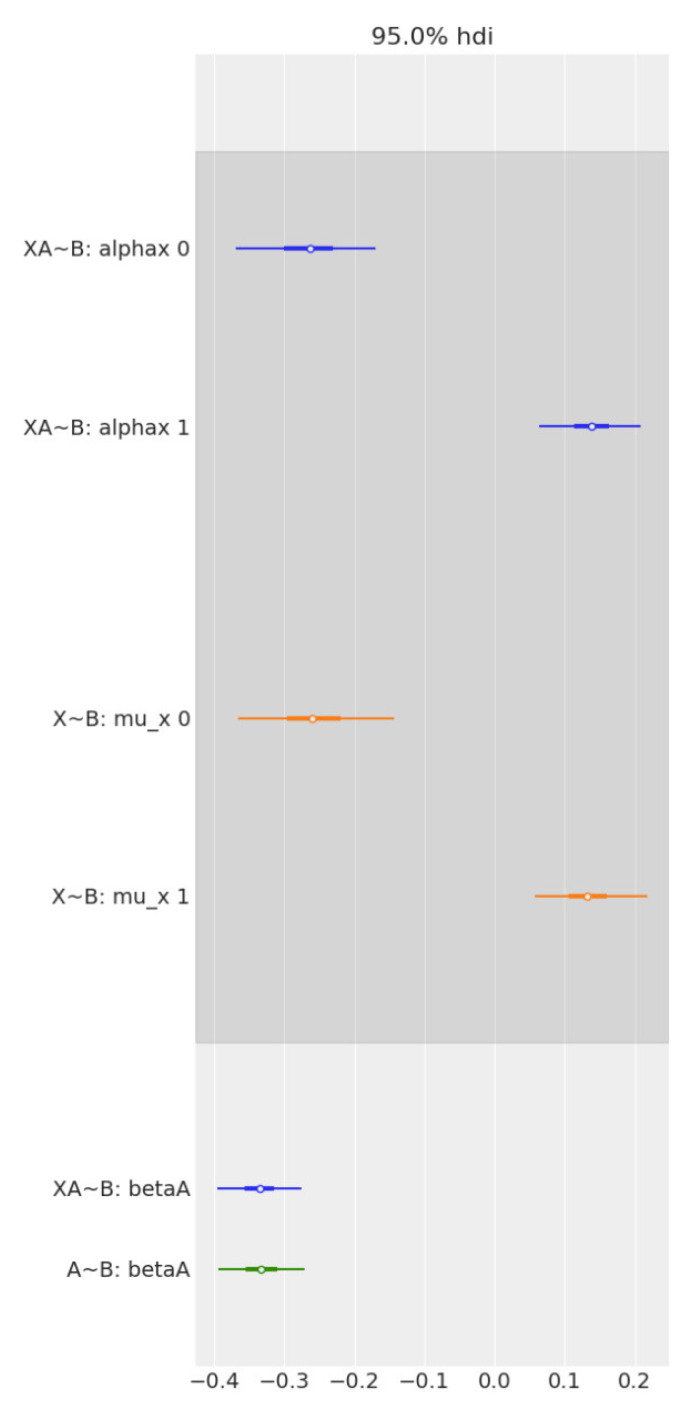
Forest plot comparing the effect of age and sex on Brain2ICV, separately and together, in multiple regression. From top to bottom, the first two bars (in blue) represent the contrast in the multiple regression model, Age + Sex → Brain2ICV. The two middle bars (in orange) represent the posterior of sex in the simple linear regression model Sex → Brain2ICV. The blue bottom bar depicts the posterior of age in the multiple regression model, Age + Sex → Brain2ICV, and the green bar depicts the posterior of age but this time for the simple regression model, Age → Brain2ICV. Once we know age (green bar at the bottom), there is no additional information derived from also knowing sex (blue bar at the bottom) because the mean and the uncertainty remain mostly unchanged. Likewise, if we know sex (orange bars), there is no significant information gain in also knowing age (blue bars at the top).

Methodologically speaking, this work departs from comparing differences between groups via point estimates and statistical testing. The pitfalls and difficulties associated with the statistical testing approach have been abundantly and convincingly described [57,58,59], and we will therefore not linger further on this point. We followed a Bayesian approach to estimate posterior probability distributions rather than point estimates. It is worth noting that under the Bayesian outlook, probabilities are tools to quantify uncertainty [60,61]. Thus, we used probability distributions to summarize the entire plausibilities of each possible value of the parameter defined in the model. For example, the posterior distribution of the mean Brain2ICV in the female group entirely lay on the positive side, so we are confident that female sex and Brain2ICV were positively associated. The opposite occurred for men, with the posterior lying entirely on the negative side, which indicates that male sex and Brain2ICV were negatively associated (Figure 5). Both observations indicate that elderly women have, on average, less brain atrophy than elderly men (Table 3).

The final goal of our methodological undertaking was to achieve a causal understanding of the factors at play in the variability of brain volume loss in ageing. To state that age causes ageing is a platitude. However, behind this innocuous statement hides one of the defining scientific challenges of our time. Is ageing inevitable, and can we devise interventions aimed at modifying the ageing process? Since we lack a theory of ageing, the question of what causes ageing and how it progresses is shrouded in uncertainty.

There are at least two main reasons for the lack of attention that causal reasoning has received in the scientific literature. One is historical and obeys reasons related to the personal preferences of leading scientists. As magisterially recounted by Judea Pearl in [45], causality was deliberately removed from statistics by Karl Pearson, who considered cause and effect animistic and unscientific concepts to be replaced by contingency tables which, in Pearson’s mind, were “the ultimate statement of the scientific description between two things” [62]. The second and most important reason for the neglect of causality is that correlations, contrary to causal conclusions, do not require a controlled experiment and are therefore easy to compute.

Although correlations have proven to be an extraordinarily successful tool to quantify pairwise relationships, correlations are lacking in situations where variables cannot be isolated. In such a scenario, we need to understand how the different variables interact with each other, which entails incorporating the direction in the association between two variables. A variable may cause another, and this cannot be accounted for with correlations, which are by definition symmetric.

The causal link between age and brain atrophy is well known, and the notion that age causes changes in brain volume, vasculature, and cognition is uncontested [50,51]. We are, however, interested in understanding whether there is an additional predictive power for Brain2ICV contained in knowing the sex if we already know the age.

Formally, in the language of causal inference, Brain2ICV is a collider, X→Z←Y (X-sex, Yage, and Z Brain2ICV). In a collider, conditioning on Z could induce a statistical association between X and Y, misleading us into thinking that age changes with sex, which is not the case. This is addressed in the multiple regression model (Supplementary Materials Equations (S1a)–(S1b)), where we quantify each effect—age on Brain2ICV and sex on Brain2ICV. We find that sex and age are conditionally independent Y ⫫ X|Z, (Y is not associated with X, after conditioning on Z) and Y ⫫ Z|X (Y is not associated with Z, after conditioning on X). Therefore, we can conclude that once we know the age of a subject, also knowing the subject’s age conveys little information in predicting their Brain2ICV.

This study is not without limitations. First, we used whole-brain segmentation data without differentiating between brain tissues or anatomical brain structures. Second, the total intracranial volume is not static throughout life, as a recent longitudinal study suggests [11]. Earlier studies also suggested that skull thickening may influence the measurement of intracranial volume [63,64]. However, Brain2ICV was herein used as a proxy for brain atrophy and not as an actual measure of brain atrophy. Brain volume loss at old age relative to young age requires the availability of MRI scans performed in both young and old age. We overcame this limitation by computing the ratio between the estimated total brain and total intracranial volumes. Thus, having only one MRI, it is possible to ascertain the diminution in brain volume within the cranium relative to the brain volume at a young age with the caveats mentioned.

## 5. Conclusions

The goal of this work was to study how brain atrophy is affected by factors such as age, the APOE gene, sex, and the level of education completed. We conceptualized the brain as a dynamic system inside a fossil container, setting the upper limit of brain volume. Accordingly, the ratio of the total brain volume to the total intracranial volume represents the percentage of the volume occupied by the brain inside the cranium. Here, this was used as a proxy of the maximum brain volume at a young age.

We found that among the variables considered in this study—age, APOE, family history of AD, sex, and school level—only age and sex affected Brain2ICV. Age was, as expected, negatively associated with Brain2ICV. The older the brain is, the smaller the ratio between the brain volume and the intracranial volume. More interestingly, we found that sex played a role in brain atrophy, with women having on average 1% larger Brain2ICV values than men. This finding is in agreement with previous works that identified sex differences in the brain during ageing and in neurodegenerative diseases. In particular, the thesis that women may have more youthful brains than men is supported by forensic and postmortem studies [5,6]. This hypothesis has been tested very recently in vivo with PET imaging, showing more persistent metabolic youth in the ageing female brain than in the male brain [65].

Part of the novelty and interest of this study relies upon its methodological underpinnings, which depart from point estimates and linear associations between variables. We used Bayesian probabilistic programming to study, in a principled way, causal inference, combining the flexibility of Bayesian probability and the applicability of sampling theory in a coherent decision-theoretical framework.

## Figures and Tables

**Table 1:** Summary of the variables used in the study: age, sex, APOE, school level, family history of AD, cognitive test score, and the estimated ratio between the brain and intracranial volumes (Brain2ICV). The mean and the standard deviation are displayed for the continuous variables, and the size of each class is displayed for categorical variables. The cognitive score is an aggregate measure of the results of the Mini-Mental State Examination (MMSE), Free and Cued Selective Reminding Test (FCSRT), Semantic Fluency, Digit-Symbol Test and Functional Activities Questionnaire (FAQ).

Variables	Mean	SD
Age	74.72	3.86
Cognitive Score	9.41	2.66
Brain2ICV	0.70	0.03
	**Total**	%
Sex		
Male	303	34.04
Female	587	65.96
APOE		
Noncarriers	726	81.57
Heterozygous *e*4	157	17.64
Homozygous *e*4	7	0.79
Level of education		
No formal education	170	19.10
Primary education	265	29.76
High school	224	25.17
University	231	25.96
Family history of AD		
No	670	75.28
Yes	220	24.72

**Table 2:** Summary of the results of the analysis of variance with a linear OLS model performed for each predictor. Both age and sex scores showed *p* values for F statistics less than the significance level of 0.01, enabling us to reject the null hypothesis (i.e., age/sex has no effect on the brain-to-ICV ratio). The APOE gene, familial AD, and the level of education completed, on the other hand, did not exhibit statistically significant effects on brain atrophy. A complete summary table of the OLS model is provided in the Supplementary Materials, Table S1 (** *p* ≤ 0.01).

	F	PR(>F)
Age	119.694	** 3.242 × 10^−26^
Sex	32.746	** 1.438 × 10^−8^
APOE	1.099	2.948 × 10^−1^
Family history of AD	1.022	3.124 × 10^−1^
Level of education	3.530	6.058 × 10^−2^

**Table 3:** The posterior distribution (*µ*, *σ* and high posterior density interval (HDI)—the shortest interval containing a given portion, e.g., 97%, of the probability density) of the model’s parameters in Equations (2a)–(2d). The first two rows present the expected Brain2ICV (the ratio between the brain volume and the intracranial volume) in each sex group (1 for men, 2 for women), the third row shows the standard deviation, and the last row denotes the expected difference in Brain2ICV between (1) men and (2) women. The contrast, *µ*_1_ − *µ*_2_, of the average Brain2ICV between men and women was always negative, which indicates that women’s brains showed less atrophy than men’s brains (*µ_2_> µ_1_*).

	Mean	sd	HDI 3%	HDI 97%
*µ*_1_	0.697	0.002	0.694	0.70
*µ*_2_	0.708	0.001	0.706	0.71
*σ*	0.027	0.001	0.026	0.028
*µ*_1_ − *µ*_2_	−0.011	0.002	−0.014	−0.007

## Data Availability

The code and data set are available at: https://github.com/grjd/causalityagingbrain (accessed on 1 September 2022).

## Supplementary Materials

The supporting information can be downloaded from: https://www.mdpi.com/article/10.3390/life12101586/s1. Table S1: Summary table of OLS model brain2icv age + sex + apoe + school level + familial AD [53]. Figure S1: On the left, the KDE of the age of the participants in the study and on the right, the volumetric estimate of the brain to ICV ratio. The skewness of the former is due to the study design which starts with 69 years old and the brain volumetric estimates follows as expected a Gaussian curve. Figure S2: Posterior distribution (left-hand) and sampling (right-hand) for parameters μ_1_, μ_2_, σ (from top to bottom, Brain2ICV mean for men, Brain2ICV mean for women and Brain2ICV standard deviation) using PyMC3 [66]. On the left, kernel density estimation (KDE) of the three parameters μ_1_, μ_2_, σ is plot for 4 parallel chains(X-axis represents the value of the parameter and the Y-axis the Frequency). On the right, the individual sampled values at each step during the sampling for the 4 chains for each parameter(X-axis represents the sample number and the Y-axis the sample value). Figure S3: The black line is a KDE of the observed data, and cyan lines are KDEs computed from each one of the 100 posterior predictive samples. The cyan lines reflect the uncertainty about the inferred distribution of the predicted data. The mean and the variance of the simulated data properly match the actual data. Figure S4: Posterior distribution (left-hand) and sampling (right-hand) for parameters α, β_A_, σ using PyMC3 [66]. On the left, kernel density estimation (KDE) plot for each of the 4 parallel chains plotted in different color run for each parameter μ_1_, μ_2_, σ (X-axis represents the value of the parameter and the Y-axis the Frequency). On the right, the individual sampled values at each step during the sampling for the four chains for each parameter(X-axis represents the sample number and the Y-axis the sample value). The slope (β_A_) is −0.33 (one standard deviation in age induces one third of change in the opposite direction in Brain2ICV) and a is as expected 0, since the data are normalized. Equations (S1a)–(S1b): that generates the data, that is to say, it requires the modeler’s input. The identification of causal associations will be consequently dependent on the model’s complexity. For example, in our case, since we are interested in the effect of Sex and Age on Brain2ICV and both predictors are conditional independent, the causal analysis is fairly simple.
