# Supplementary material for: A Causal Analysis of the Effect of Age and Sex Differences on Brain Atrophy in the Elderly Brain
Source: Life (Basel). 2022 Oct 12;12(10):1586. doi: 10.3390/life12101586 (PMC9656120; doi:10.3390/life12101586)
Supplement: Supplementary file 1 [file life-12-01586-s001.zip › life-1887516-supplementary material-1.pdf]

I. Supplementary Material

Figure S1 shows the distribution of age and of the Brain to ICV. The brain to ICV is Gaussian and the age distribution reflects the experiment design which limited the age to participate to 69 or older.

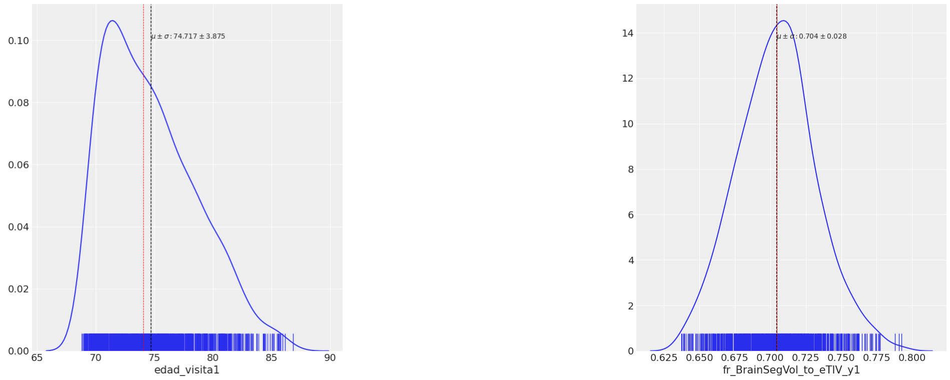

(a) (b)

Figure S1: On the left, the KDE of the age of the participants in the study and on the right, the volumetric estimate of the brain to ICV ratio. The skewness of the former is due to the study design which starts with 69 years old and the brain volumetric estimates follows as expected a Gaussian curve.

Table S1 shows the summary table of the OLS model *brain2icv age* + *sex* + *apoe* + *school level* + *familial AD* with the values normalized.

Figure S2 shows graphically the posterior distribution and the sampling for the three unobserved variables in the model (μ_1, μ_2, σ). On the left-hand of Figure S2, we show the kernel density estimation of the marginal distributions of each parameter and on the right hand we show the individual sampled values. The right-hand of Figure S2 plots the individual sampling values.

Figure S2 left side suggests that males as suggested by the posterior average (μ_1) get into older age with more brain volume loss or less Brain to intracranial ratio than women (μ_2)). For a better visualziation of the difference between brain loss relative to intracranial volume, see Figure S3a. The normalized average for males μ_1 is negative and positive for females μ_2. Figure S3a also indicates that the high posterior density interval (HDI) is slightly wider males.

Since we have computed the posterior, we can use it to simulate data to assess the predictive quality of the model by comparing how consistently the simulated data match the observed data. Figure S3b shows the *posterior predictive checks* which allows us to evaluate the model by comparing the observed data and the model predictions (100 posterior predictive samples). The figure shows a good match between the mean and the variance of the simulated data and the actual data.

Figure S4 shows graphically the posterior distribution and the sampling for the three unobserved variables in the model ($Age \to Brain2ICV$). The variable Brain2ICV, B_i, is the observed data and therefore we do not need to sample its values. On the left-hand of Figure S4, we show the kernel density estimation of the marginal distributions of each parameter and on the right hand we show the individual sampled values.

It is, however, worth recalling that causal analysis requires to postulate upfront the process

Table S1: Summary table of OLS model *brain2icv   age + sex + apoe + school level + familial AD* [53].)

**Dep. Variable:**	fr_BrainSegVol_to_eTIV_y1	**R-squared:**	0.153
**Model:**	OLS	**Adj. R-squared:**	0.148
**Method:**	Least Squares	**F-statistic:**	31.82
**Date:**	Sat, 04 Jul 2020	**Prob (F-statistic):**	7.27e-30
**Time:**	20:24:27	**Log-Likelihood:**	2008.2
**No. Observations:**	890	**AIC:**	-4004.
**Df Residuals:**	884	**BIC:**	-3976.
**Df Model:**	5		

	**coef**	**std err**	**t**	**P> |t|**	**[0.025**	**0.975]**
**Intercept**	0.8805	0.017	52.363	0.000	0.848	0.914
**edad_visita1**	-0.0024	0.000	-10.940	0.000	-0.003	-0.002
**sexo**	0.0105	0.002	5.722	0.000	0.007	0.014
**apoe**	-0.0022	0.002	-1.048	0.295	-0.006	0.002
**familial_ad**	0.0020	0.002	1.011	0.312	-0.002	0.006
**nivel_educativo**	-0.0015	0.001	-1.879	0.061	-0.003	6.81e-05

**Omnibus:**	22.145	**Durbin-Watson:**	1.960
**Prob(Omnibus):**	0.000	**Jarque-Bera (JB):**	25.003
**Skew:**	0.328	**Prob(JB):**	3.72e-06
**Kurtosis:**	3.494	**Cond. No.**	1.48e+03

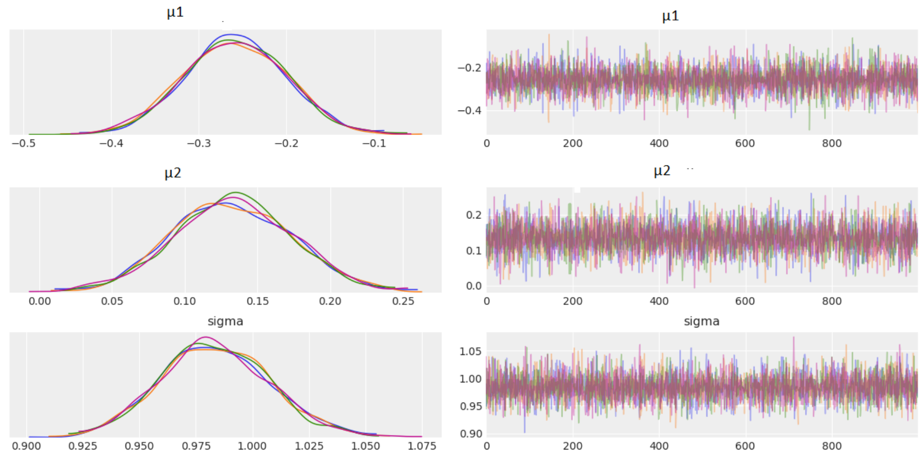

Figure S2: Posterior distribution (left-hand) and sampling (right-hand) for parameters μ_1, μ_2, σ (from top to bottom, Brain2ICV mean for men, Brain2ICV mean for women and Brain2ICV standard deviation) using PyMC3 [66]. On the left, kernel density estimation (KDE) of the three parameters μ_1, μ_2, σ is plot for 4 parallel chains(X-axis represents the value of the parameter and the Y-axis the Frequency). On the right, the individual sampled values at each step during the sampling for the 4 chains for each parameter(X-axis represents the sample number and the Y-axis the sample value).

that generates the data, that is to say, it requires the modeler's input. The identification of causal associations will be consequently dependent on the model's complexity. For example, in our

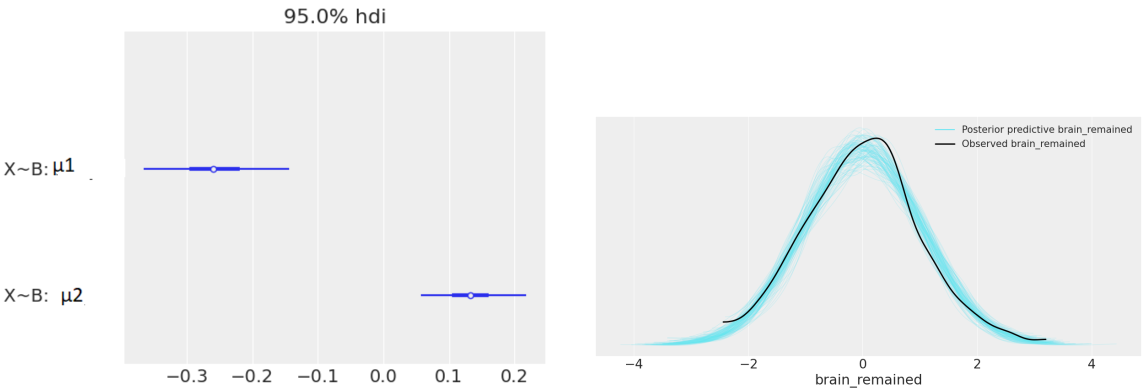

Figure S3: The black line is a KDE of the observed data, and cyan lines are KDEs computed from each one of the 100 posterior predictive samples. The cyan lines reflect the uncertainty about the inferred distribution of the predicted data. The mean and the variance of the simulated data properly match the actual data. On the left side, the plot that compares the posterior of the parameters μ_1 (Males) and μ_2 (Females). The mean of the Brain2ICV in females is 0.133 and -0.259 for males, the standard deviation for males (0.058) is larger than in females (0.041). On the right side, The black line is a KDE of the observed data, and the cyan lines are KDEs computed from each one of the 100 posterior predictive samples. The cyan lines reflect the uncertainty about the inferred distribution of the predicted data. The mean and the variance of the simulated data properly match the actual data.

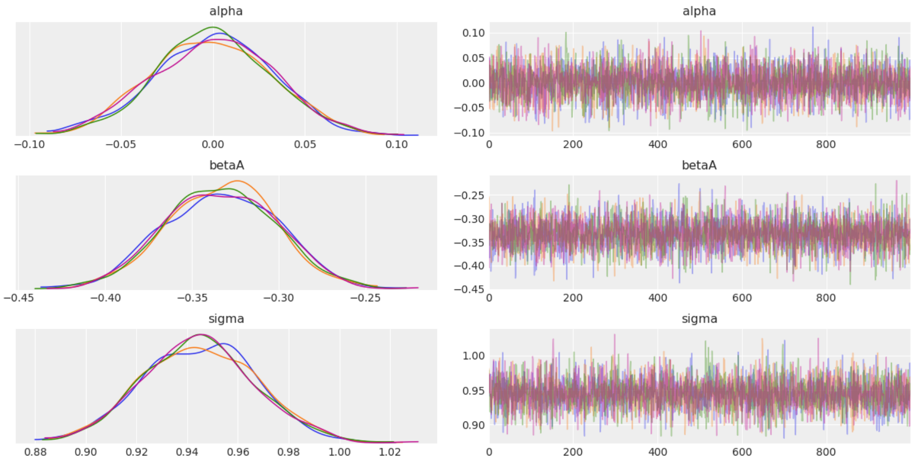

Figure S4: Posterior distribution (left-hand) and sampling (right-hand) for parameters α, β_A, σ using PyMC3 [66]. On the left, kernel density estimation (KDE) plot for each of the 4 parallel chains plotted in different color run for each parameter μ_1, μ_2, σ (X-axis represents the value of the parameter and the Y-axis the Frequency). On the right, the individual sampled values at each step during the sampling for the four chains for each parameter(X-axis represents the sample number and the Y-axis the sample value). The slope (β_A) is -0.33 (one standard deviation in age induces one third of change in the opposite direction in Brain2ICV) and α is as expected 0, since the data are normalized.

case, since we are interested in the effect of Sex and Age on Brain2ICV and both predictors are conditional independent, the causal analysis is fairly simple.

$$B_i \sim N(\mu_i, \sigma) \tag{S1a}$$

$$\mu_i = \alpha + \beta_A A_i + \gamma_{X[j]} \tag{S1b}$$

$$\alpha \sim N(0, 1) \tag{S1c}$$

$$\beta_A \sim N(0, 1) \tag{S1d}$$

$$\gamma_{X[j]} \sim N(0, 1), \textit{for j=1,2} \tag{S1e}$$

$$\sigma \sim HalfNormal(1) \tag{S1f}$$

where B_i denotes the variable Brain2ICV, γ_j represents the average of Brain2ICV for $(j = 1)$ male and $(j = 2)$ female and the prior distribution of σ is half-normal. A_i is the Age of subject i. Since all three variables are standardized, we expect the intercept α and the parameter β_A to be around zero.
